# A new method to induce nonalcoholic steatohepatitis (NASH) in mice

**DOI:** 10.1186/s12876-019-1041-x

**Published:** 2019-07-15

**Authors:** Feryal Savari, Seyyed Ali Mard, Mohammad Badavi, Anahita Rezaie, Mohammad Kazem Gharib-Naseri

**Affiliations:** 1 Physiology Research Center (PRC), Department of Physiology, School of Medicine, Ahvaz Jundishapur University of Medical Sciences, Ahvaz, Iran; 2 Persian Gulf’s Physiology Research Center (PRC), Research Center for Infectious Diseases of Digestive System (Alimentary Tract Research Center), Department of Physiology, School of Medicine, Ahvaz Jundishapur University of Medical Sciences, Ahvaz, Iran; 3 Department of Pathobiology, School of Veterinary Medicine, Shahid Chamran University of Ahvaz, Ahvaz, Iran

**Keywords:** NAFLD, NASH, Fructose, High fat diet, Western diet, Cigarette smoke, Mice

## Abstract

**Background:**

General overnutrition is one of the key factors involved in the development of nonalcoholic fatty liver disease (NAFLD) as the most common liver disease occur by two steps of liver injury ranges from steatosis to nonalcoholic steatohepatitis (NASH). Here the effect of fructose, fat-rich and western diet (WD) feeding was studied along with aggravative effect of cigarette smoking on liver status in mice.

**Methods:**

Sixty-four male NMRI mice were included in this study and assigned into 4 groups that fed standard, fructose-rich, high fat-, and western-diet for 8 weeks and then each group divided in two smoker and nonsmoker subgroups according to smoke exposing in the last 4 weeks of feeding time (*n* = 8). Histopathological studies, serum biochemical analyses and hepatic TNF-α level were evaluated in mice to compare alone or combination effects of dietary regimen and cigarette smoking.

**Results:**

Serum liver enzymes and lipid profile levels in WD fed mice were significantly higher than in other studied diets. Exposing to cigarette smoke led to more elevation of serum biochemical parameters that was also accompanied by a significant increase in hepatic damage shown as more severe fat accumulation, hepatocyte ballooning and inflammation infiltrate. Elevated TNF-α level confirmed incidence of liver injury.

**Conclusion:**

The finding of this study demonstrated that a combination of cigarette smoke exposure and WD (rich in fat, fructose, and cholesterol) could induce a more reliable mouse model of NASH.

## Background

Over the last several decades, there is increasing concern about the rising daily exposure to cigarette smoke along with hyper-calorie intake on human health [1–3]. Actually, this global change in dietary habits is likely to contribute to the generation of intrahepatic lipid subsequently, renders the hepatocytes susceptible to a variety of damages that increased the probability of disease progression to more severe conditions, including non-alcoholic steatohepatitis (NASH) [1, 4, 5]. NASH is the more perilous form of NAFLD characterized by development of hepatocellular injury, ballooning and inflammation in the steatotic hepatocyte [6].

A large number of animal models have been provided in the context of the evaluation of onset and progression of NASH, but along with their advantages, currently models have several limitations [7, 8]. Therefore, there is a need to induce an appropriate model that is similar to human disease with respect to causes trigger NASH, and also considering the interferences of the same environmental risk factors are involved in the progression of disease in human [7, 9]. According to the current pathogenic factors especially dietary factors, that have been lead to disease development and progression and matches modern lifestyle, a reliable disease model should be induced by diet and not by administration of chemical toxins and/or genetic manipulation [7, 9].

Insulin resistance (IR) is one of the main metabolic disturbances linked to NAFLD/NASH and previously established as an independent predictor of NASH [10–15]. It could be used to distinguish NASH from simple steatosis especially when has accompanied with elevated serum alanine transaminase ALT and gamma glutamyl transferase (GGT) [11, 16–19].

Tumor necrosis factor α (TNF-α) as a main inflammatory mediators, has also reported to predict NAFLD development [20, 21].

Cigarette smoking has been pointed as one of the main environmental possible factor contributing disease progression [22–24]. However, despite increasing researches during the last decades, no data is available regarding the potential influence of cigarette smoking (Cs) on the course and severity of western dietary model of NAFLD.

Starting from this background, the aim of our study was to report the development of a novel combination mouse model for NASH in setting of a western diet and animal exposure to Cs, with an emphasis on the comparison of different dietary components effects on the histopathological and biochemical changes of disease.

## Methods

### Animals grouping and dietary interventions

Sixty-four male NMRI mice (25–30 g), 60 days of age, were purchased from the animal house of Ahvaz Jundishapur University of Medical Sciences (AJUMS, Ahvaz-Iran) and were housed in a controlled environment (temperature 22 ± 2 °C, 12 h light and dark cycles). All experiments were conducted in accordance with the Guide for the Care and Use of Laboratory Animals [25]. AJUMS’s Experimental Animals Ethics Committee approved all protocols (IR.AJUMS.REC.1395.409).

Mice were randomly divided into 4 groups, consisting of 16 animals per group and were fed with four different types of diet for 8 wks. Control group consisted of animals on standard chow diet (SD); Fructose animals group (Fru), were fed a chow diet and received 30% fructose in drinking water; HFD animals group were fed a diet high in fat (30% beef tallow) supplemented with 30% fructose; and WD animals group were fed a Western diet for 8 wks. The Western diet was obtained mixing 0.4% cholesterol, 30% beef tallow to triturated SD diet and also supplemented with 30% fructose. In the last 4 weeks of feeding period (weeks 5–8 of the study), half of the mice in each group were exposed to cigarette smoke 4 cigarettes daily, 5 days a week for 4 weeks as smoker subgroups. Nonsmoker mice underwent the same procedure but exposed only to fresh air. All animals had ad libitum access to their respective drink and food at all times.

### Cigarette smoke exposure

To create a smoke environment, a smoking apparatus was designed with nine compartments linked by holed partitions. A peristaltic pump was applied to smoke the cigarette with suction. Finally, the cigarette smoke that get from a commercial brand containing 9‌mg total particulate matter (TPM) and 0.7 mg nicotine per cigarette redirected into central compartment of chamber and no animal was placed on it. Each animal’s exposure lasted 15 min with 5 min smoke-free interval for ventilation by system uncovering under a hood [26]. This smoking procedure was repeated 4 times a day, 5 days per week for 4 weeks [27].

All mice were fasted overnight and sacrificed under ketamine and xylazin deep anesthesia (80 + 6 mg kg ^− 1^, i.p, respectively) following blood extraction via cardiac puncture and liver removal [28]. Blood samples were collected and centrifuged at 6000 g for 10 min. Separated serum subjected to the determination of the levels of aspartate transaminase (AST), ALT, alkaline phosphatase (ALP), GGT, total bilirubin (TBL), glucose, insulin, triglyceride (TG), total cholesterol (TC), low-density lipoprotein (LDL), and high-density lipoprotein (HDL) [29]. Livers were removed immediately, weighed and fixed in 10% formalin solution for histopathological evaluations and TNF-α level measurement.

### Histopathological analysis

#### Liver histology

Liver samples were processed routinely in 10% neutral buffered formalin for 24 h, and then embedded in paraffin. The fixed liver tissues sliced into 5 μm thickness and stained with hematoxylin and eosin (H&E) [30].

#### Pathology, staging of NAFLD

Hematoxylin and eosin staining was performed to evaluate liver histology and grading liver injury using a scoring system for NASH established by Kleiner et al. and Brunt [28, 31]. The outcomes of different dietary feedings on the development of steatosis and NASH were blindly assessed by an expert liver pathologist according to this scoring system for semi-quantifying of histological changes.

According to this standardized scoring system, the sum of steatosis (0–3), lobular inflammation (0–3), and hepatocellular ballooning degeneration (0–2) scores considered to be the NAFLD activity score (NAS) (score of 0–2: not NASH; 3–4: borderline; 5–8: NASH).

According to proposed criteria, scores were as follows: steatosis grade, 0: less than 5%, 1: between 5 and 33%, 2: between 33 and 66%, and 3: more than 66%.

Cell ballooning scored as none (0), mild (few swelled cells; 1) and severe (many swelled cells; 2) based on its severity.

For lobular inflammation, minimal or absence of inflammatory cells accumulation (infiltration) scored as grade 0, mild infiltration (grade 1), moderate to severe infiltration (grade 2) and severe inflammatory cells accumulation (grade 3).

Altogether, diagnosis of NASH is dependent on histological findings of steatosis along with hepatocellular ballooning and lobular inflammation without requirement of fibrosis appearance [32].

#### Biochemical parameters measurements

The level of ALT, AST, ALP and GGT as biomarkers of liver injury, concentration of TG, TC, and HDL, fasting serum glucose and TBL were determined biochemically using related commercial kits (Pars Azmoon; IR Iran), by a serum autoanalyzer (BT- 1500-A-A, Rome, Italy). LDL-C was calculated as total cholesterol – (HDL-C + triglyceride /5). Insulin levels of serum was evaluated using ELISA assays kits and HOMA-IR (homeostasis model assessment-estimated insulin resistance) was calculated according to the following formula: fasting insulin (μIU/dL) × fasting glucose (mg/dL)/405 [33].

#### Determination of TNF-α level

The level of TNF-α in supernatant of liver homogenate were measured by commerical ELISA kit, according to manufactures’ instruction on the basis of the Biotin double antibody sandwich technology (BT, China).

#### Statistical analysis

Results were expressed as means ± standard deviation (SD) and statistical significance of *P* < 0.05. One-way analysis of variance (ANOVA) with tukey’s post hoc tests were used for the identification of significant differences for multiple comparisons among dietary groups. Kruskal–Wallis followed by mann-whitney’s test was used to analyze histopathologic scoring.

## Results

Effect of different diets feeding on serum glucose, insulin and lipid profile.

As shown in Table 1, increased TC and TG concentrations have been found in both subgroups of HFD, and WD fed mice (*P* < 0.001) as well as in smoker fructose fed mice (*P* < 0.05).

**Table 1:**
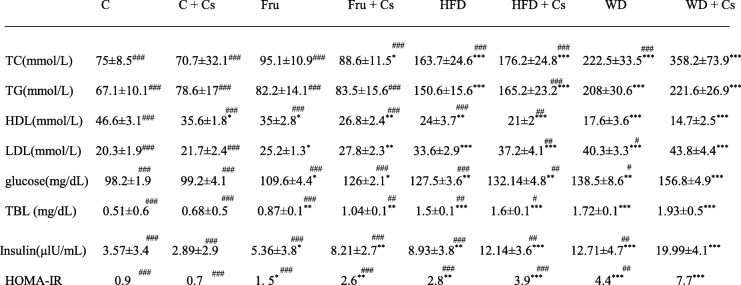
The comparative effects of different diets on metabolic parameters after 8 weeks

Data are expressed as means ± SD, *n* = 8 in each group. ^*^P < 0.05, ^**^P < 0.01, and ^***^P < 0.001 significant difference compared to mice fed standard diet as control. ^#^P < 0.05, ^##^P < 0.01, and ^###^P < 0.001 significant difference compared to smoker mice fed WD

Serum concentration of TG and TC in the nonsmoker subgroups did not differ from those of mice exposed to cigarette. In two subgroups of HFD and WD (*P* < 0.001) and smoker subgroup of fructose fed mice (*P* < 0.01), serum level of LDL significantly increased. Elevation of LDL level in both smoker and nonsmoker mice fed WD was significantly higher than other groups (P < 0.05 and P < 0.001; respectively). The serum level of HDL significantly decreased in all studied groups compared to control (P < 0.05, *P* < 0.01, and *P* < 0.001).

WD + Cs group had significantly higher levels of Glu, TBL, insulin and IR index (HOMA-IR) than their other groups. The GGT level also remarkably elevated in these mice.

### Effect of different diets feeding on liver functional enzymes

In all groups of mice, different diets feeding led to significant elevations of AST, ALT, and ALP levels in serum compared to control (*P* < 0.01, *P* < 0.001 in all cases). Cigarette exposure in control mice also led to significant elevations of ALP (*P* < 0.05) and AST (P < 0.05). The results also demonstrated that, smoker sub-group of animals had significantly higher levels of ALT and AST than both control and their non-smoker subgroup mice (*P* < 0.05, *P* < 0.01, *P* < 0.001). As demonstrated in Table 2, the most elevation of ALT, AST, and ALP were appeared in smoker subgroup of WD fed mice compared to other smoker and nonsmoker fed mice (P < 0.001 in all cases). The serum level of GGT also revealed a significant elevation in other groups with more severity in WD + Cs as compared to control (P < 0.05, P < 0.01, P < 0.001).

**Table 2:**
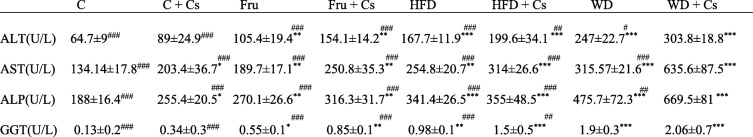
The comparative effects of different diets on serum level of hepatic enzymes after 8 weeks

Data are expressed as mean ± SD, n = 8 in each group. ^*^P < 0.05, ^**^P < 0.01, and ^***^P < 0.001 significant difference compared to mice fed standard diet as control. ^#^P < 0.05, ^##^P < 0.01, ^###^P < 0.001 significant difference compared to smoker mice fed WD

### Effect of different diets feeding on histopathological alterations

**Fig. 1:**
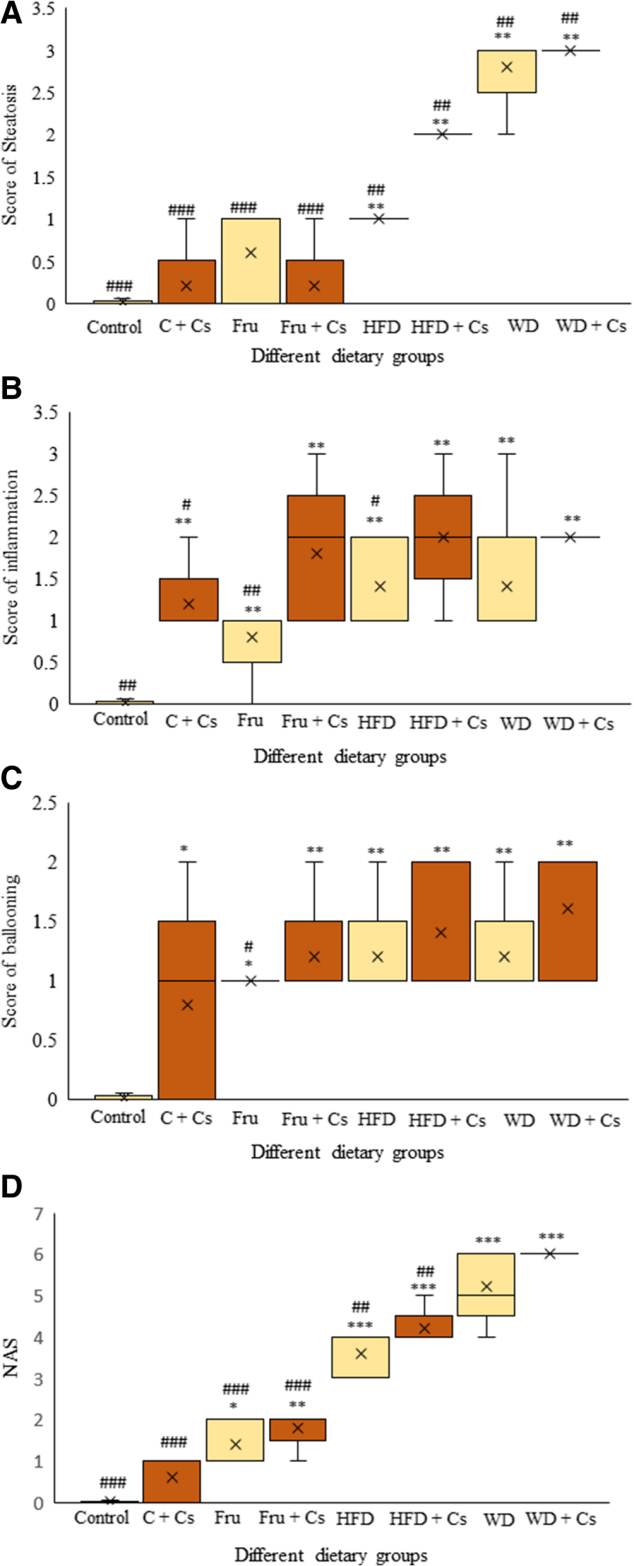
The comparative effects of different diets on liver histology to evaluate fatty change (**a**), inflammation (**b**), ballooning degeneration (**c**) (kruskal-wallis followed by mann-whitney’s test), and NAS score (**d**) (one-way ANOVA followed by tukey’s test) in hepatic tissues of mice fed with SD, Fru, HFD, and WD with or without cigarette exposure after 8 weeks with hematoxylin and eosin staining using the semiquantitative NAS System. Data are depicted using box and whisker plots showing median, minimum and maximum values, n = 8 in each group. ^*^P < 0.05, ^**^P < 0.01 and ^***^P < 0.001 significant difference compared to mice fed standard diet as control. ^#^P < 0.05, ^##^P < 0.01 and ^###^P < 0.001significant difference compared to smoker mice fed WD. **c**: control; Cs: Cigarette smoke; Fru: fructose; HFD: high fat diet; WD: western diet; NAS: NAFLD Activity Scoring

As shown in Fig. 1, livers of control mice exhibited histologically normal structure. In exposed subgroup of control mice, moderate cell swelling represented ballooning degeneration (grade 1) along with mild inflammation (grade 1) has been found without any fatty change (grade 0).

Fructose fed subjects failed to display any signs of obvious appearance of steatosis (grade 0) but showed mild level of inflammation and ballooning degeneration (grade 1). Exposure to smoke increased inflammatory scoring to 2–3 grade with mild degree of hepatocyte ballooning (grade 1) but fatty change was not found. It should be noted that two mice in this subgroup had shown amyloidosis in addition to inflammation that marked as uniform pink sediments located in hepatic sinusoidal wall. The nature of amyloid sediments was confirmed by Congo red staining which became orange.

Upon high fat diet feeding for 8 weeks, fatty changes in hepatocytes were observed along with mild to moderate cell ballooning (grade 1) mainly localized in the periportal area. In smoker subjects, nearly most of hepatocytes developed steatosis as grade 2 with greater extent of inflammation (grade 2) and hepatocyte ballooning (grade 1).

**Fig. 2:**
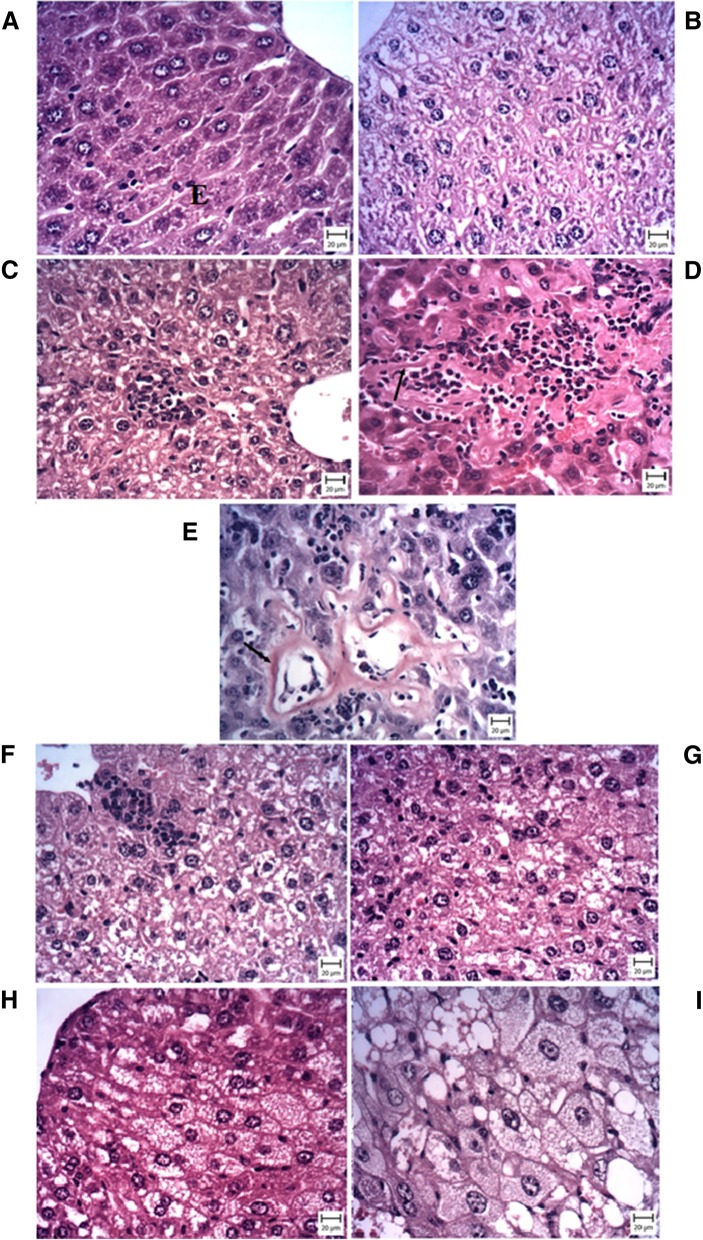
Representative microscopic images (Bar: 20 μm) of H&E stained liver sections from Control (**a**), Control + CS (**b**), Fructose fed diet (**c**), Fru + Cs (**d**, **e**: Congo Red), HFD fed (**f**), HFD + Cs (**g**), WD fed (**h**), WD + Cs (**i**) mice show histopathological changes including: fatty change, hepatocyte ballooning (as indicated by arrow) and inflammatory cell infiltration. Fru + Cs (E) image represents congo-red stain of liver section

In western diet fed mice, more severe steatohepatitis characterized by extensive micro- and macro-vesicular steatosis (score 3) with more prevalence and intensity of inflammation (grade 2) and nearly mild degree of hepatocellular ballooning (grade 1) by end of feeding duration (Fig. 2).

The exposing animals to smoke led to more severe inflammation (grade 3) and ballooning degeneration.

### Effect of different diets feeding on hepatic TNF-α level

**Fig. 3:**
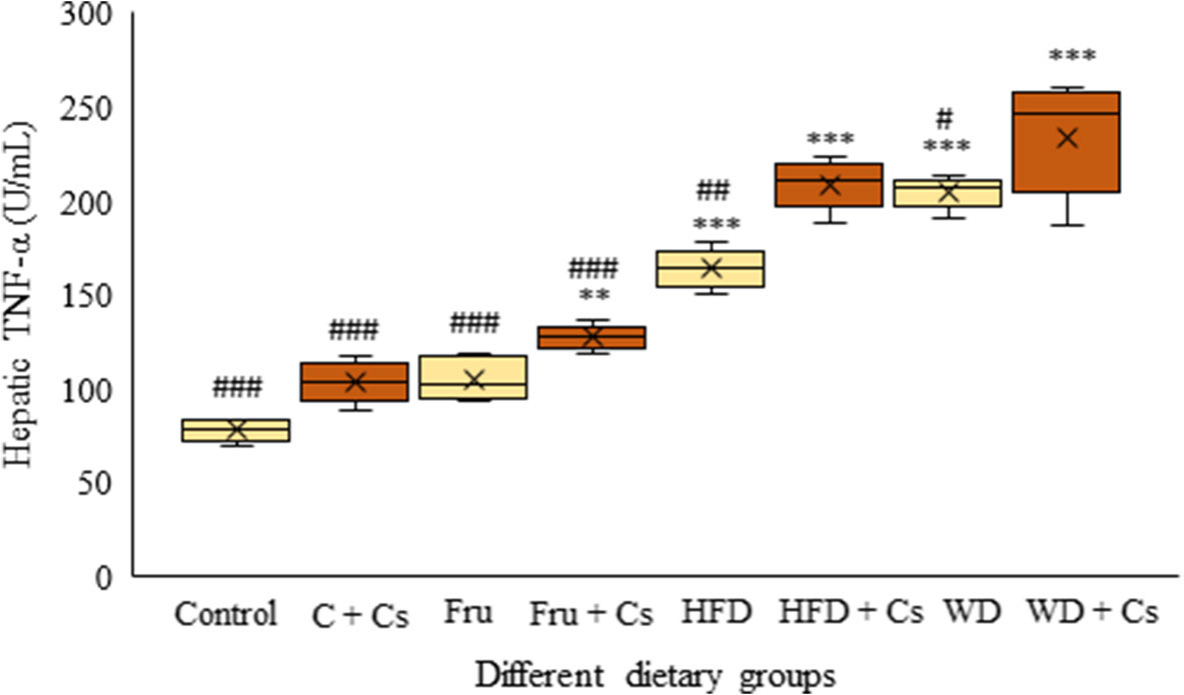
The comparative effects of different diets on hepatic inflammatory cytokine level, TNF-α after 8 weeks**.** Data are depicted using box and whisker plots showing median, minimum and maximum values, n = 8 in each group. ^*^P < 0.05 and ^***^P < 0.001 significant difference compared to mice fed standard diet as control. ^#^P < 0.05 and ^###^P < 0.001significant difference compared to smoker mice fed WD. C: control; Cs: Cigarette smoke; Fru: fructose; HFD: high fat diet; WD: western diet; TNF-α: tumor necrosis factor- α

As shown in Fig. 3, we reached to considerable increase of TNF-α level in WD + Cs mice when compared to the control group (*P* < 0.001). Cigarette exposure led to increase TNF-α level in mice livers (*P* < 0.05). After 8 weeks of feeding, TNF-α level was significantly higher in livers of exposed mice fed fructose, high-fat diet and western diets when compared to non-smoker mice fed related diet (P < 0.05, *P* < 0.01). Moreover, there was a trend for TNF-α levels to be higher in diets plus Cs than in control mice (P < 0.05, P < 0.001).

## Discussion

An appropriate experimental model of NASH could reflect the pathologic aspects of human disease and resembles the histological and etiological pattern of disease [34, 35].

In the present study, we outlined a mouse model to determine the effects of caloric excess and damaging role of cigarette smoking to induce model of NAFLD that progress to NASH and attempted to compare three different dietary patterns provided as diet enriched with fructose, fat or western diet.

The presence of insulin resistance along with dyslipidemia, as manifestations of metabolic syndrome, enhance possibility of NASH induction. Moreover, elevated serum levels of GGT and also ALT could be important for correlating these metabolic disorders to NAFLD progression [11, 17, 18].

Our data showed that fructose feeding failed to induce significant dyslipidemia but fasting serum glucose and insulin modestly elevated.

Mice HFD diet exhibited mild increase of biochemical parameters and more severity of these changes have been found in mice on WD diet which reflects significant IR and metabolic disturbance. Substantial increase of GGT activity confirmed existence of metabolic disturbance which linked IR to NASH. Therefore, it seems that co-elevation of ALT, GGT and especially IR index, could be used to confirm NASH induction as found in WD + Cs mice [36, 37]. This finding is in line with previous reports suggesting the existence of IR in NAFLD subjects and with more severe in NASH [38].

Elevation of ALT, AST, ALP and GGT that would be reflects incidence of hepatocellular injury [39], has been found in all dietary groups and higher levels were achieved in their smoker subgroups. These changes were more pronounced in the WD + Cs subjects. Increased serum value of TBL also found to exhibit hepatic damage [40]. However, histological changes are still final validity of liver abnormalities in this context [32, 41, 42].

In our study, hepatic changes were highly different depending on the diet content and smoke exposing. As showed our comparative histopathology of hepatic tissues, fructose intake failed to induce obvious steatosis but diet rich in fat was able to change liver status and it was more severe in mice fed western diet. These damaging effects were found to be exacerbated after cigarette exposing (Fig. 1a). Increasing NAS scoring in HFD and WD dietary regimens exposed to cigarette smoke as 5; and 6 respectively reflected the occurrence and development of NASH phenotypes progressed from borderline to more intensive NASH model (Fig. 1d).

Given that inflammatory response plays a critical role in the pathogenesis of NAFLD, we next evaluated the incidence of inflammation that could be demonstrated by TNF-α level elevation. The significantly higher inflammation characterized by inflammatory infiltrate and elevated hepatic TNF-α level was observed in mice on WD + Cs. Our results to show increased level of hepatic TNF-α were in consistent with its main role in NAFLD/NASH pathogenesis and confirmed histopathological evidences [43–45].

Somewhat contrary to previous research [1, 3, 28, 46–48] that used fructose rich diet, alterations observed at the level of the hepatocytes were only as hepatocellular ballooning with minor signs of fatty change that not enough to confirm fatty liver histologically (Fig. 1c).

Increase of hepatic fat uptake lead to initial diminish of TG serum level as a mechanism of TG accumulation in the liver [37]. Consistent with previous findings [49, 50], in our experience, the circulating levels of TG was also observed as decreasing in some analyzed serum results that has pointed to signs of initial complications of hepatic fatty change.

Potential effect of HFD in NASH model induction has been shown [51]. However, just mild steatosis and minor signs of inflammation achieved in our study (Fig. 1). Variable and even inconsistent results with regard to the degrees and patterns of NASH histological features have been reported from previous [3, 51, 52].

Various results that have been reported in this context might be related to the fact that not only the extra energy receiving but also other factors such as duration of feeding, content, and composition of dietary fat may be involved in NASH induction [8]. Susceptibility to NASH especially in diet-induced NAFLD models provided notable evidence to document the role of strain as well as animal’s gender on the NAFLD developing [53, 54]. Furthermore, strain differences may affect hepatic reaction heterogeneously so that some strain such as C57BL/6 mice [55] are prone to develop metabolic and histopathological features of NAFLD, while others only exhibit some metabolic changes and cannot be observed any liver disease feature [50]. Our study could induce NAFLD progression model (NASH) in NMRI mice.

As supported by experimental and epidemiological studies, high-fat diets involved in the onset of hepatic steatosis while high carbohydrate diets attributed to promote of the steatosis development [56]. Therefore, these finding together concluded that a combined patterns of feeding has been able to show stronger effect on the liver phenotype characterizes NASH [3, 32] though its development needs to longer time [28].

In the present study, mice were fed with Western-type diet, showed significantly more pronounced fat deposition accompanied with developed cell degeneration and inflammation which represented disease severity enhancement, similar to a previous study [32, 56, 57].

Cigarette smoke has been introduced as a cofactor that worsen the severity of NAFLD but it seems that cannot able to induce disease features alone [24, 58–60]. Except for elevation of liver enzymes activity and related histopathological changes, cigarette smoke failed to affect liver in context of hepatic and/or serum lipid profile in intact animals [58, 61].

These changes were also achieved in smoker subgroups of fructose fed subjects with greater changes but still no evidence of obvious lipid droplet deposition was found. It could be concluded that no fructose diet nor cigarette smoke was enough to induce NASH.

The cigarette exposing subgroup of HFD showed early signs of steatohepatitis. These signs were more pronounced features in smoker subgroup of WD as expected, and was accompanied with enhancement of NAS scoring.

Fast food diet as a more closely dietary pattern in human which load to NASH, requires long period of administration time and will face difficulties for researching [49, 62]. Considering the exacerbating effects of cigarette smoking on NAFLD progression, we designed a comparative study on mouse model of NASH according to combined pattern of interventions have nowadays converted to habits of modernized life style in shorter frame of time.

Furthermore, expected NASH features with severe steatosis, hepatocellular ballooning, and inflammatory infiltration along with metabolic and biomolecular changes were found in livers of cigarette exposed mice fed WD.

## Conclusion

Taken together, the important finding of the present study was the ability of western diet to induce NASH model in mice and smoke exposing was found to worsen the damaging effect of high calorie intake. Applying a combination of involved possible factors in NAFLD progression led to introduce an applicable model of NASH induction in shorter frame of time that may be provide a more functional tool for evaluation of NAFLD pathogenesis.

## Data Availability

The data that support the findings of this study are available from (Feryal Savari (first author)) who is writing her thesis based on the current study, so are not publicly available as of now.

## Abbreviations

ALP: Alkaline phosphatase
ALT: Alanine transaminase
ANOVA: Analysis of variance
AST: Aspartate transaminase
C: Control
Cs: Cigarette smoking
Fru: Fructose
H&E: Hematoxylin and eosin
HDL: High-density lipoprotein
HFD: High- fat diet
LDL: Low-density lipoprotein
NAFLD: Nonalcoholic fatty liver disease
NAS: NAFLD activity score
NASH: Nonalcoholic steatohepatitis
SD: Standard deviation
SD: Standard diet
TC: Total cholesterol
TG: Triglyceride
TPM: Total particulate matter
WD: Western diet
